# Cardiac magnetic resonance characteristics of acute anthracycline-induced cardiotoxicity

**DOI:** 10.1186/1532-429X-18-S1-P268

**Published:** 2016-01-27

**Authors:** Olga H Toro-Salazar, Joanna Ferranti, Glenn S Slavin, Kan N Hor

**Affiliations:** 1 Connecticut Children's Medical Center, Hartford, CT USA; 2 GE Healthcare, Silver Spring, MD USA; 3 Nationwide Children's Hospital, Columbus, OH USA

## Background

Cardiac magnetic resonance imaging (CMR) is the gold standard for quantification of global and regional myocardial function and is able to detect subclinical myocardial dysfunction in the setting of a wide variety of myocardial disease processes, including anthracycline induced cardiomyopathy (AIC). Preliminary studies using T2-weighted sequences have demonstrated increase in signal intensity suggestive of myocardial edema during cancer therapy. We hypothesized that reductions in mid-wall peak circumferential (εcc) and longitudinal (ειι) strain magnitude and increase in T2 relaxation suggestive of myocardial edema would precede changes in EF in a cohort of pediatric patients newly diagnosed with cancer studied at baseline and after 24-48 hours at set intervals of anthracycline cumulative dose up to maximal therapy.

## Methods

Twenty subjects aged 10-22 years, diagnosed with cancer that required anthracycline therapy, were identified and prospectively enrolled between January 2013 and November 2014. Seven subjects withdrew and 43 visits were completed for the remaining 13 subjects. All subjects underwent CMR with routine cine acquisition, tissue characterization, left ventricular ejection fraction (EF) and strain analysis using a modified 16-segment model.

## Results

**Figure 1:**
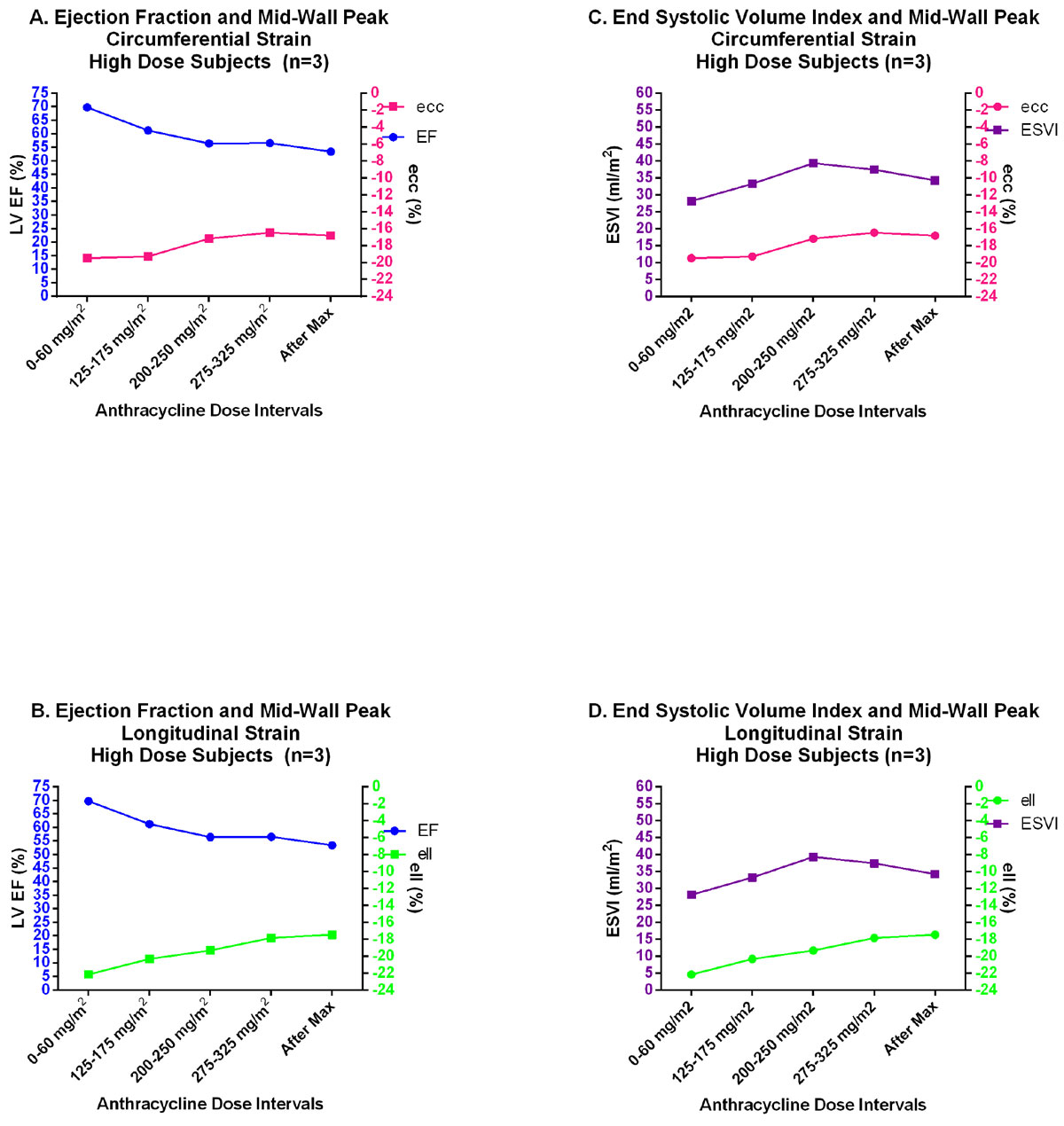
Figure 1

**Figure 2:**
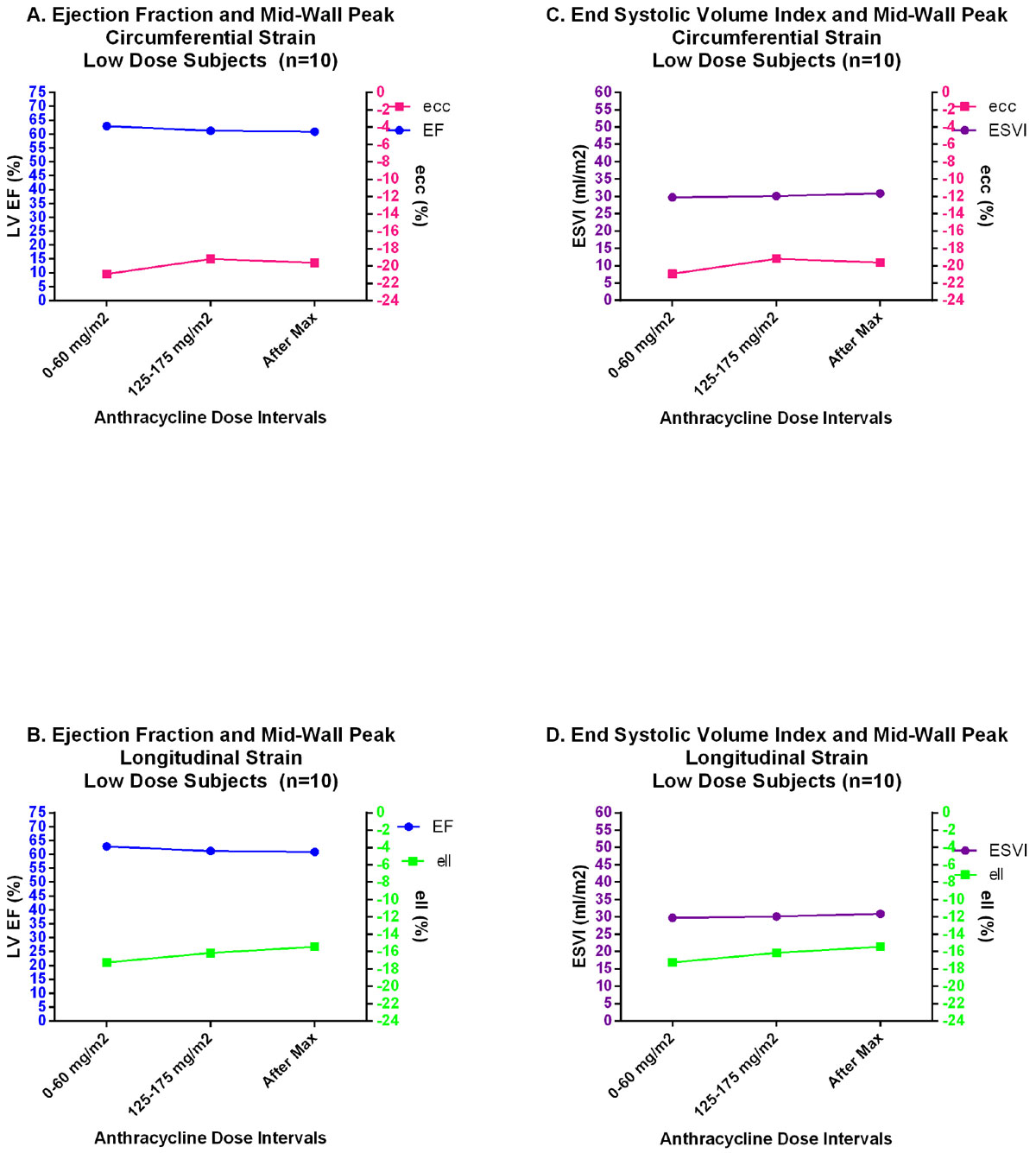
Figure 2

Patients in the high dose group demonstrated an increase in LV end-systolic volume index (ESVI) with progressive decline in ejection fraction (EF), and strain magnitude overtime (Figure 1 a-d). Decline in ειι (−17.2 ± 1.0) occurred at lower cumulative doses (200-250 mg/m^2^) followed by a decline in εcc (−17.82 ± 2.6) at 275-325 mg/m^2^ and EF < 55% at doses >375 mg/m^2^. No significant increase in T2 relaxation or late gadolinium enhancement (LGE) was present in any of the study subjects. Among the low dose group there were similar changes up to maximal cumulative dose of 200 mg/m^2^ (Figure 2 a-d). Subjects in the low dose group with the lowest ειι at baseline attained the lowest EF in follow-up.

## Conclusions

Asymptomatic pediatric patients exposed to high dose anthracycline therapy developed abnormal strain parameters at lower cumulative doses, with a decline in ειι occurring earliest, when compared to changes in EF. With the exception of one high dose patient an increase in myocardial T2 relaxation or positive LGE were not observed acutely after anthracycline therapy. The excellent reproducibility for LVEF and early decline in strain magnitude by CMR may help identify patients at high risk for development of AIC and guide therapy to prevent or decrease the incidence of late cardiac events.

